# Effect of bee venom on IL-6, COX-2 and VEGF levels in polycystic ovarian syndrome induced in Wistar rats by estradiol valerate

**DOI:** 10.1186/1678-9199-19-32

**Published:** 2013-12-12

**Authors:** Latifeh Karimzadeh, Mohammad Nabiuni, Homa Mohseni Kouchesfehani, Hamed Adham, Amir Bagheri, Azar Sheikholeslami

**Affiliations:** 1 Department of Cell and Molecular Biology, School of Biological Sciences, Kharazmi University, Karaj, Iran

**Keywords:** Polycystic ovarian syndrome, Honeybee venom, Interlukin-6, Cyclooxygenase-2, Vascular endothelial growth factor

## Abstract

**Background:**

Polycystic ovarian syndrome (PCOS) is a low-grade inflammatory disease characterized by hyperandrogenemia, hirsutism, chronic anovulation and vascular disorder. Interleukin-6 (IL-6), cyclooxygenase-2 (COX-2) and vascular endothelial growth factor (VEGF) are triggered by inflammatory stimuli and lead to angiogenesis and pathogenesis of the ovary. Honeybee venom (HBV) contains an array of biologically active components possessing various pharmaceutical properties. This study was designed to assess the possibility of HBV application as an anti-inflammatory therapeutic agent to suppress levels of the main inflammatory mediators IL-6, COX-2 and VEGF.

To induce PCOS, 1 mg of estradiol valerate (EV) per 100 g of body weight was subcutaneously (SC) injected into eight-week-old rats. After 60 days, 0.5 mg/kg of HBV was administered Intraperitoneal (IP) for 14 consecutive days, and the results of PCOS treatment were investigated. Rats were then anesthetized with CO2, and the ovaries were surgically removed. Serum IL-6 was detected by the ELISA kit. Immunoexpression of COX-2 and VEGF were examined in three groups: EV-induced PCOS, HBV-treated PCOS and control animals.

**Results:**

Thickness of theca layer, number and diameter of cysts and levels of IL-6 significantly decreased in HBV group relative to PCOS group. The immunohistochemical analysis showed an increase in COX-2 and VEGF expression in PCOS group whereas HBV-treated rats presented weak and irregular immunostaining.

**Conclusions:**

Our results suggest that the beneficial effect of HBV may be mediated through its inhibitory effect on serum IL-6 level and ovarian COX-2 and VEGF expression.

## Background

Polycystic ovarian syndrome (PCOS), one of the most common causes (70%) of anovulatory infertility, affecting 6% of women of reproductive age, is introduced as a low-grade chronic inflammation [1]. Even though the term PCOD is used for this disorder, PCOS is more common, due to its heterogeneous clinical signs such as hirsutism, hyperandrogenism and ovarian histopathological disorders. Histological features of ovaries in PCOS include the presence of more than 12 follicles smaller than 10 mm, surrounding the stroma, and an increase in the thickness of follicular theca and the ovarian stroma volume, which may be due to neoangiogenesis and vascular endothelial growth factor (VEGF) expression in the ovary [1,2].

VEGF is a mediator of the cyclical growth of blood vessels that occurs in the female reproductive tract [3,4]. Furthermore, VEGF is widely distributed in normal and malignant tissue and is highly expressed in areas of active vascular proliferation [5]. Unlike other growth factors, the mitogenic activity of VEGF is restricted to vascular endothelial cells. Increased expression of VEGF has been described recently in hyperthecosis of polycystic ovaries [2,6].

Cyclooxygenase-2 (COX-2) causes a decline in prostanoid biosynthesis and is involved in inflammation, cell growth, and specialization. COX-2 is induced by mitogens, growth factors, cytokines, and tumor promoters [7]. Human diseases that exhibit proliferative activity, inhibition of apoptosis and neoangiogenesis have shown COX-2 overexpression [8]. COX-2 plays an important role in inflammation by prostaglandin synthesis. Nonsteroidal anti-inflammatory drugs (NSAIDs) are anti-inflammatory agents acting through the inhibition of cyclooxygenase [7]. Moreover, the great importance of COX-2 in PCOS is apparent from its proliferative effect on the theca layer cells of the ovary, where ovulation occurs. The direct and indirect roles of COX-2 (through TNF-alpha) in the thickening of the theca layer of the ovary have been reported [9]. We demonstrated that COX-2 was overexpressed in PCOS rats [10]. However, the role of COX-2 overexpression during mammalian ovary cycles is still less well defined. These findings correspond with a common evolutionary background for PCOS, metabolic and inflammatory disorders.

Angiogenesis induced by either endogenous COX-2 or exogenous prostaglandins (PGs) is accompanied by increased expression of VEGF [11]. VEGF expression is up-regulated by COX-2-mediated PGs [12]. Treatment of the cells overexpressing COX-2 with a COX-2-selective inhibitor also decreased PGE2 level and attenuated VEGF expression [13].

Interleukin-6 (IL-6) is produced mostly by macrophages and also by adipocytes. In PCOS patients circulating levels of tumor necrosis factor-alpha (TNF-alpha), IL-6, C-reactive protein (CRP), as well as white blood cells (WBCs) and neutrophils have been found to be elevated compared with controls [14-16].

Bee venom is comprised of a large number of pharmaceutical components, most notably melittin, apamin, adolapin and peptide 401, which have been widely investigated to reveal their physiological effects, and to discover their compatibility with different anti-inflammatory mechanisms. The reduced expression of COX-2 and phospholipase (PL) A_2_ and the decreased levels of tumor necrosis factor alpha (TNF-alpha), IL-1, IL-6, nitric oxide (NO) and reactive oxygen species (ROS) are suggested as being associated with the anti-inflammatory effects of HBV in some tissues [17-20].

In the present study, we have provided evidence for a direct involvement of inflammation in the maintenance and progression of PCOS. Clinical observations also suggest that PCOS is associated with inflammation and proliferation of circulating inflammatory molecules such as IL-6, COX-2 and VEGF. According to the anti-inflammatory effects of HBV on arthritis and some of the inflammatory diseases, we hypothesize that HBV decreases the incidence of PCOS, as an inflammatory disease. To test this hypothesis, firstly we evaluated the serum IL-6 levels, ovarian COX-2 and VEGF expression in normal rats; and then to assess the close relationship between PCOS, systemic inflammation and metabolic syndrome, we compared these factors in normal rats with PCOS animals; and finally, to discover the anti-inflammatory effects of HBV, we investigated alterations in these inflammation indexes, and eventually, the hormonal and histological changes of ovary in HBV-treated rats compared with PCOS ones.

## Methods

Adult female Wistar rats weighing 170 ± 20 g (7–8 weeks of age) from the animal house of the Kharazmi University, Tehran, Iran, were kept in a central animal care facility, housed in plastic cages (30 × 19 × 13 cm) under a 12-hour light, 12-hour dark cycle (lights on from 6:00 to 20:00). Humidity and temperature were set at 55 ± 15% and 20 to 24°C, respectively, and free access to water and commercial food (Behparvar Com., Iran) was provided. All procedures were carried out according to the Guidelines for the Care and Use of Laboratory Animals (National Research Council, 1996).

In this experiment, adult female Wistar rats with a 2–3 regular estrous cycle period within a twelve- to fourteen-day period were used. PCOS rats were selected on the basis of displaying a minimum of two continuous estrous cycles. Rats were in the estrous stage of their reproduction cycle.

Iranian honeybee venom was collected from *Apis mellifera* by means of an electric shocker apparatus composed of a shocker and a collector unit. The shocker unit produces a light electric shock once every few seconds. Honeybees were stimulated with light electric shock and sting in beehives. The collector unit is a network of wires with small gaps and a glass plane between them. Every 25 minutes, the shocker unit turned off and the dried bee venom material on collector panel was collected by scraping.

Initially, animals were divided into three groups: controls (no injection), PCOS [2 mg injection SC of estradiol valerate (Aburaihan Co., Iran)] and sham 1 (a similar dose of sesame oil with no estradiol content), with n = 8 for all groups. All animals were under vaginal smear analysis for a period of 60 days until the appearance of persistent vaginal cornification (PVC), a sign of follicular cysts in the ovary. After verifying the induction of PCOS, the PCOS group was divided into two subgroups, PCOS and PCOS + HBV. PCOS + HBV received 0.5 mg/kg HBV IP for 14 consecutive days, while PCOS (i.e., sham 2) group received physiological saline solution. Sham 1 and sham 2 groups were later removed from the experiment due to their lack of any difference with the control.

At around 9:00 am rats were anesthetized with CO_2_, trunk blood was collected, and serum samples were separated by centrifugation at 6,000 rpm for five minutes. Samples were kept at −40˚C for later serological experiments. Fatty tissue was separated under a loop microscope. Ovaries were separated from the twisted oviduct tubes. Ovarian samples for immunohistochemical experiments were fixed in formalin, embedded in paraffin, sectioned, and mounted on glass slides. Twelve serial sections (5-mm thickness) from each sample were prepared for immunohistochemistry.

### IL-6 assay

Serological analysis was performed to measure serum IL-6 levels and hormonal alterations. In order to detect serum IL-6, an ELISA kit (rat IL-6 platinum ELISA®, Bender Medsystems, Austria) was used according to the manufacturer’s instructions. The assay was performed in triplicate according to the manufacturer’s recommended procedures. The results were expressed as mean ± SD (pg/mL) of three individual rats. The sensitivities of the assay for IL-6 were 12 pg/mL.

### Immunohistochemistry

Sections of 5 mm in thickness were cut from formalin-fixed tissue embedded in paraffin blocks and mounted onto coated slides. Sections were de-waxed in xylene and rehydrated in a graded alcohol series (100, 90, 70 and 50%). After deparaffinization, sections were boiled in citrate buffer (0.05 M) in a microwave oven to reveal antigens. Endogenous peroxidase was quenched with 3% (v/v) hydrogen peroxide (ten minutes at room temperature). Samples were rinsed three times for five minutes in PBS, and nonspecific binding was blocked with dehydrated nonfat milk (50 mg/mL diluted in PBS).

Thereafter, tissue sections were rinsed three times with 0.05% PBST ween-20 (PBS-T) and then incubated overnight at 4˚C with rabbit and goat polyclonal antibody for VEGF and COX-2 (1:1000 and 1:500 dilution, Abcam, UK). After being washed four times with PBS-T, sections were incubated with anti-rabbit and anti-goat secondary antibody (Bethyle Laboratories, Inc., USA, and Universal LSAB™ + Kit/HRP, Rabbit/Mouse/Goat, Product n K0690) for 90 minutes, at 1:300 dilution and 37˚C to amplify the signal. Following three washes in PBS, sections were incubated with immunoreactivity complexes detected by 3, 3’-diaminobenzidine tetrahydrocholoride (Immunohistochemistry Accessory Kit, Bethyl Laboratories Inc., USA). Slides were then counterstained with Mayer’s hematoxylin and mounted on crystal (entellan). Negative controls were included in each experiment by incubating tissue sections with antibody dilution buffer instead of the primary antibody. Positive control slides consisted of rat hippocampus cells for COX-2 and vessels for VEGF.

### Immunohistochemical evaluations

The H-score is the sum of the proportion of cells showing different degrees of reactivity. For H-score assessment, ten fields were chosen at random at 400× magnification and the staining intensity of each slide was scored as 0, 1, 2 or 3 corresponding to the presence of negative, weak, intermediate or strong brown staining, respectively. The total number of cells in each field and the number of cells stained at each intensity were counted. The average percentage positive was calculated and the following formula was applied:

H ‒ score = [(% of cells stained at intensity category 1) × 1] + [(% of cells stained at intensity category 2) × 2] + [(% of cells stained at intensity category 3) × 3]

An H-score between 1 and 300 is obtained, in which 300 is equal to 100% of cells stained strongly. The raw data were converted to Quick score values by multiplying the quantity and staining intensity scores.

### Statistical analysis

All statistical analyses were performed with INSTAT version 3.0 software. The differences in immunohistochemistry and area of positive staining were evaluated using the analysis of variance (ANOVA). Data were expressed as means ± standard error (SEM), and the results were taken from at least three independent experiments, performed in triplicate. Values of p of 0.05 or less were considered statistically significant.

## Results

In addition to a significant increase in body weight of the PCOS group, an increase in adipose tissue of the abdominal cavity was macroscopically detected. On the other hand, the decreases in both abdominal adipose tissue and total body weight detected macroscopically in the HBV-treated group were not significant [21].

A significant increase in the weight of the ovaries was detected in the PCOS group compared to controls. Furthermore, the HBV-treated group presented a significant decrease in ovary weight compared to PCOS.

Chemiluminescent immunoassay (CLIA) showed a significant increase in the levels of both testosterone and estradiol in the PCOS group. However, progesterone levels did not decrease significantly. These three hormones were diminished in animals treated with bee venom. The reduction observed in testosterone and estradiol levels in HBV-treated animals were significant. Progesterone levels differed significantly between the HBV-treated compared to the polycystic group, which was due to formation of corpora lutea in the ovaries of the former. These data, in particular the increase in androgen (i.e., testosterone) levels, demonstrate the success of PCOS induction, and that bee venom was able to reduce estradiol and testosterone levels.

A significant decrease was observed in the number of primary follicles, antral follicles, corpora lutea, primordial follicles and preantral follicles in PCOS ovaries. In addition, some large cystic follicles with a thin granulosa of 2–3 cell layers were observed. In this group, no corpus luteum, as a sign of ovulation, was detected. In the sham group, ovaries contained no cyst but several follicles at different stages of development, and also corpora lutea, were distinguishable. These results were indicative of a complete induction of the PCOS phenotype. In rats treated with HBV, the number of primordial and preantral follicles and corpora lutea increased, whereas the number of cysts decreased significantly compared to the sham group. In addition, some corpora lutea were observed in the HBV group, which was considered a sign of relative improvement in PCOS ovaries [10].

### IL-6 assay

**Figure 1:**
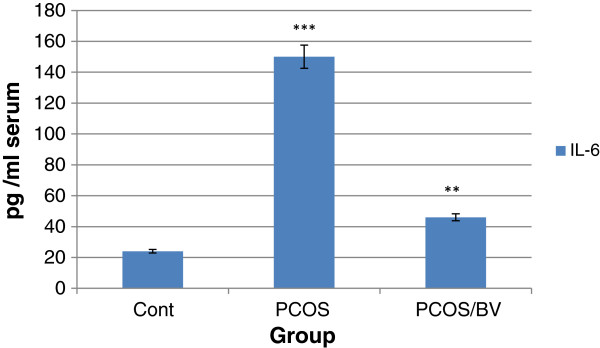
**Honeybee venom treatment effects on levels of IL-6 production (pg/mL) in polycystic ovarian syndrome (PCOS).** Baseline parameters of PCOS rats (n = 8), control (n = 8) and bee venom-treated rats (n = 8). *** p < 0.001 ** p < 0.01 vs. control.

In this study, PCOS induction led to a significant rise in IL-6 inflammatory index (p < 0.001 vs. control rats). The effect of HBV (0.5 mg/kg) on the level of IL-6 in PCOS rats was examined for 14 days after complete induction of PCOS. As shown in Figure 1, the respective IL-6 levels in control, PCOS and HBV-treated PCOS rats were 24, 150 and 46 pg/mL. The IL-6 level in HBV-treated rats was reduced by p < 0.01 vs. PCOS rats. Results showed that administration of HBV (0.5 mg/kg) significantly reduced the IL-6 level in comparison with that in the PCOS group (Figure 1).

### VEGF and COX-2 expression

**Figure 2:**
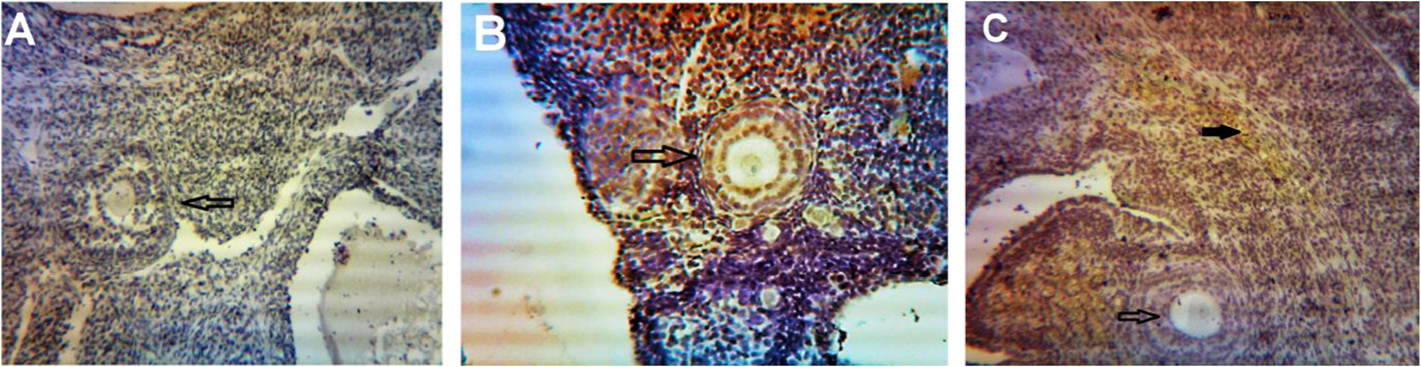
**Expression of COX-2 in primary and secondary follicles. (A)** normal ovary; **(B)** strict expression of COX-2 is observable in these follicles in PCOS group; **(C)** COX-2 expression is restricted to blood vessels and ovary stroma (filled arrow) in HBV group. Magnification 100 × .

**Figure 3:**
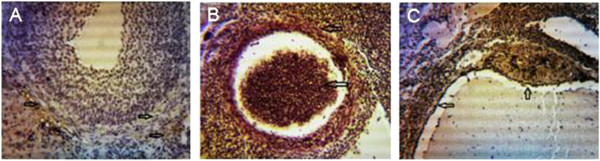
**Expression of COX-2 in infolded layers and follicular liquid of Graafian follicles and cysts. (A)** Low expression of COX-2 is seen in granulosa and theca layers in control group. **(B)** Due to high levels of angiogenesis, the density of follicular liquid, as well as COX-2 expression are high. The thick theca and granulosa layers indicate high expression levels of COX-2 in PCOS group. **(C)** A cyst is distinguishable, with no decrease in expression levels of COX-2 in HBV group. Arrows depict the predominant location of COX-2. Magnification 100 × .

**Figure 4:**
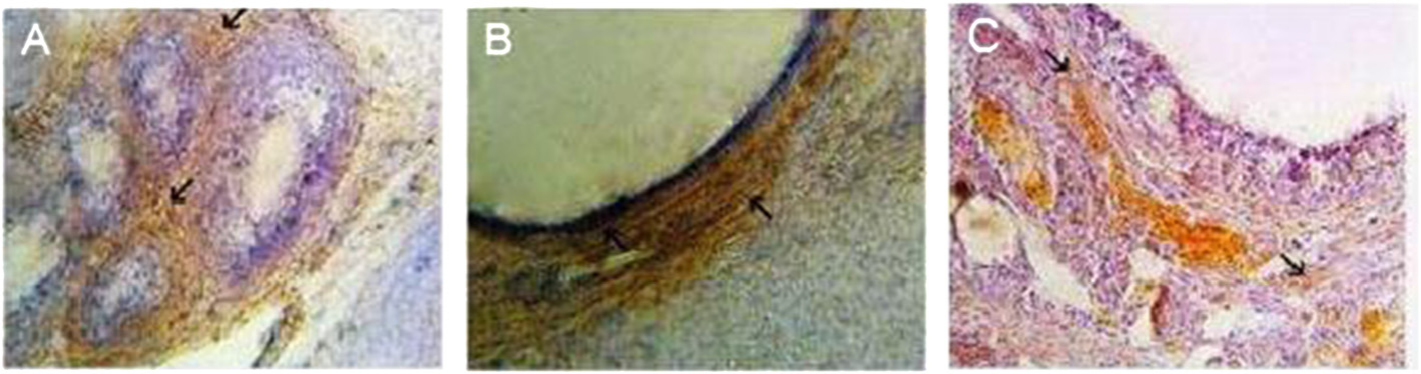
**Expression of VEGF in infolded layers and follicular liquid of Graafian follicles and cysts. (A)** VEGF is highly expressed in theca layer of ovaries, whereas, this high expression is not seen in granulosa layer of antral follicles in control group. **(B)** High expression of VEGF is observable in granulosa and theca layers in PCOS group. **(C)** VEGF is just expressed in theca layer, especially in blood vessels of venom-treated ovaries. Arrows depict the predominant location of VEGF in theca layer. Magnification 100 × .

**Figure 5:**
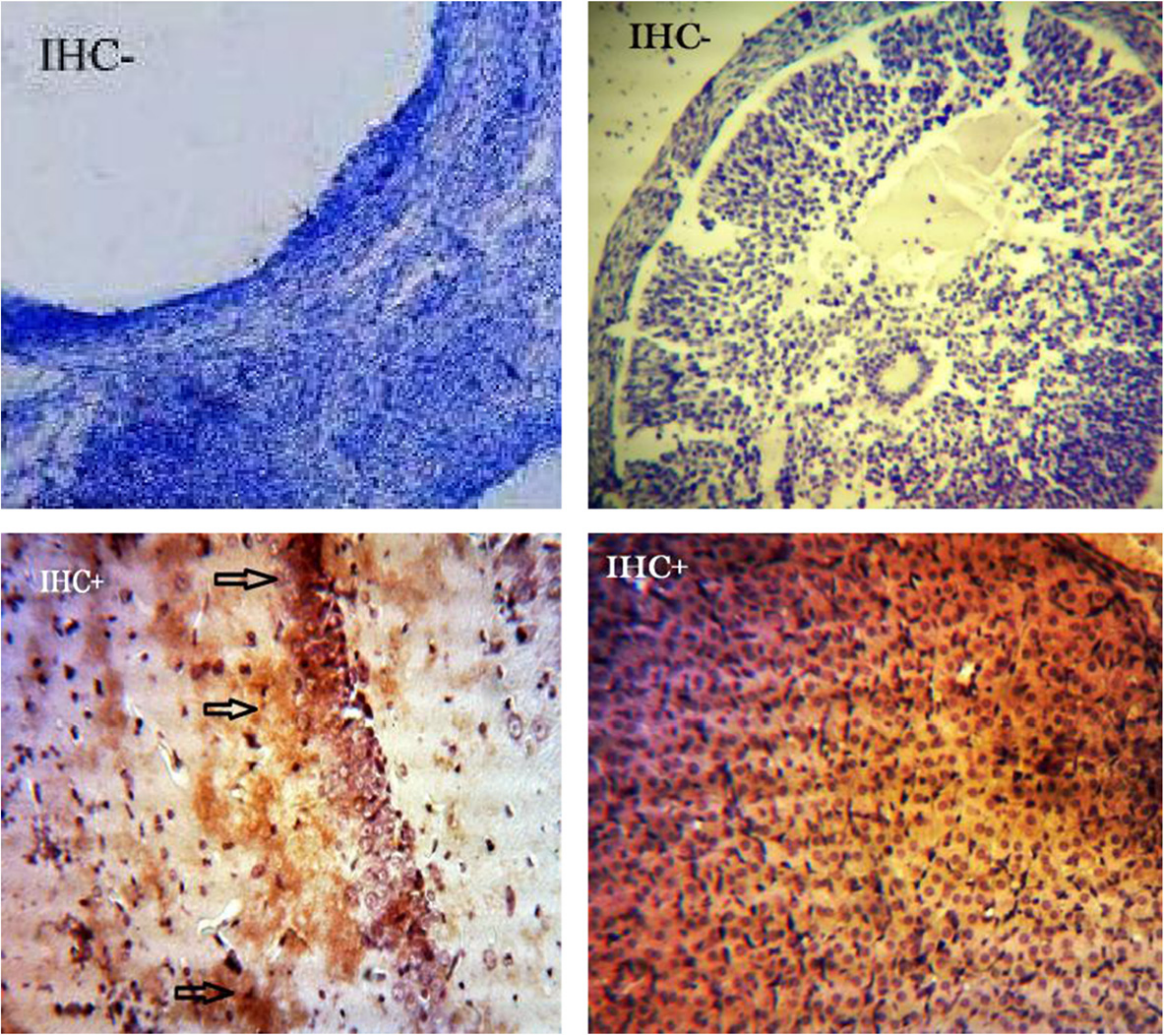
**Negative and positive controls for COX-2 and VEGF.** Negative controls were included in each experiment by incubating tissue sections with antibody dilution buffer instead of the primary antibody (antral follicle cells). Positive control slides consisted of rat hippocampus cells for COX-2 (left panel) and vessels of corpus luteum for VEGF (right panel).

**Figure 6:**
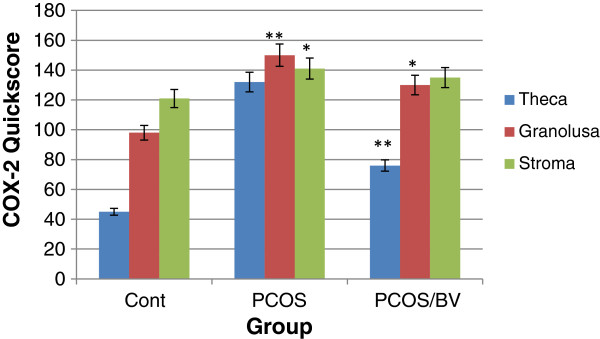
**Quick score analysis of COX-2 immunostaining in control, PCOS, and HBV-treated ovary.** Results were expressed as mean ± SEM from n = 8. ***p < 0.001, **p < 0.01, *p < 0.05 vs. control.

**Figure 7:**
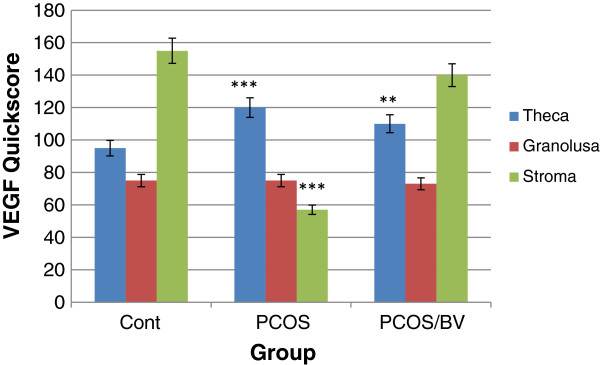
**Quick score analysis of VEGF immunostaining in control, PCOS, and HBV-treated ovary.** Results were expressed as mean ± SEM from n = 8. ***p < 0.001, **p < 0.01 vs. control.

Expression of VEGF protein was detected by immunohistochemistry in all of the specimens examined. The PCOS group presented strong immunoreactivity to COX-2 and VEGF was observed in theca layers and consequently in follicular fluid, and some VEGF expression was seen in the granulosa layer, although not as consistently as in theca cells. Diffuse, usually weak expression was seen in ovarian stroma, within individual cells. COX-2 and VEGF expression in stromal cell in the HBV-treated PCOS group was stronger than in the PCOS group. But immunostaining in the theca layer in HBV-treated PCOS group was less intense than in PCOS group. In the HBV-treated PCOS group, COX-2 presented low expression in granulosa and theca layers in preantral follicles and no expression in the primary and secondary follicles. These staining patterns were similar to the ovaries of healthy control group, in which VEGF expression was limited to stroma and theca layer and COX-2 expression limited to granulosa and theca layer of Graafian follicle (Figures 2, 3, 4, 5, 6, 7).

## Discussion

PCOS is a consequence pathway between IL-6, CRP, TNF-alpha, androgens, COX-2 and VEGF: PCOS, as a proinflammatory state, is evidenced by elevated plasma concentrations of a number of inflammatory mediators such as IL-6 [16,22]. IL-6 stimulates C-reactive protein synthesis in the liver [23]. Consequently, CRP induces secretion by p38 MAPK-TLR4 signal pathway in rat vascular and adipose cells [24,25]. According to an investigation by Spaczynski *et al.*[26], TNF-alpha causes proliferation and differentiation of theca cells and augmentation of steroidogenesis of follicular layer cells. Additional migration of mononuclear cells (MNC) into adipose tissue in PCOS models and their differentiation into macrophages (releasing other cytokines) in stroma visceral parts result in the activation of adipocyte TNF-alpha production [27].

TNF-alpha induces insulin resistance in endothelial cells and activates NF-kB/NFIL6/CRE through PKC/MAP kinase/JNK/P38 signaling pathways and thereupon provokes an increase in the expression of cyclooxygenase 2 (COX-2) [28-30]. Invading macrophages up-regulate COX-2 and prostaglandin E_2_ (PGE_2_) suggesting that PGE_2_ may affect macrophage function via autocrine or paracrine mechanisms. Colocalization of IL-6 with COX-2 was frequently observed. PGE_2_ has been shown to facilitate cell survival and induce the production of IL-6 in peritoneal macrophages *in vitro*[8].

The elevated expression of COX-2 and VEGF in theca layer of ovary causes an augmentation in the diameter of this layer, due to their ability to stimulate cell proliferation, angiogenesis and to provoke a decrease in ovulation [2,6,10].

Androgens can lead to increased levels of lipolysis and free fatty acids. Free fatty acids are primary ligands for toll-like receptors, which are central regulators for innate immune responses. Therefore, free fatty acids act as direct links between hyperandrogenism and inflammation [14]. This is the reason why we can consider cytokine expression (IL-6) as an important factor in PCOS as a low-grade chronic inflammation disease [16,22]. Thus, we infer that increasing androgens as a confirming sign of PCOS led to increases in IL-6, COX-2 and VEGF, which we have considered.

In PCOS, hyperandrogenism and hyperglycemia may be capable of promoting inflammation. They are able to generate reactive oxygen species (ROS) from peripheral blood mononuclear cells. ROS-induced oxidative stress activates nuclear factor κB (NFκB), which is involved in expression of COX-2 and induction of IL-6 [27,31]. Furthermore, the association of plasma inflammatory mediators with circulating androgens can contribute significantly to the promotion of PCOS.

With regard to an increase in serum IL-6 overexpression of tissue COX-2 and VEGF as inflammatory signs in our PCOS rats, PCOS may be analyzed considering its inflammatory and metabolic aspects. If we consider PCOS as an inflammatory disorder, we can decrease androgens and the incidence of PCOS mediated by the bee venom.

Various mechanisms have been reported in recent studies on anti-inflammatory and/or anti-arthritis action of HBV and its components [17,18,32]. The anti-arthritis effects of melittin, an HBV constituent, are suggested to be decrease in COX-2 and phospholipase A2 expression and decline in the levels of TNF-alpha, IL-1, IL-6 and ROS. Anti-inflammatory activity has also been reported by adolapin, an HBV constituent, in carrageenan-induced edema, polyarthritis rats and PG-induced rat inflammation [32]. Inhibiting the PG synthesis system through COX-2 inhibitory properties is considered to be the action mechanism of adolapin. COX-2 activity and COX-2 mRNA expression are strongly inhibited by HBV in a dose-dependent manner, and present no cytotoxic effects. The inhibitory effect of HBV was comparable to indomethacin, a well-known COX-2 inhibitor [19]. It is also reported that HBV is probably an effective RA modulator, thus hindering the protease activity and ROS removing. HBV indirectly decreases the expression of COX-2 by reducing the amount of TNF-alpha, whereas TNF-alpha activates NF-κB/NFIL6/CRE by PKC/MAP kinase/JNK/P38 signaling pathways and consequently provokes an increase in COX-2 expression. C-reactive protein and VEGF levels were significantly lower in groups given HBV therapy compared to the control group [10,32,33].

Kim *et al*. [32] speculated that HBV produced a decreased incidence of arthritis via its inhibitory effect on immune responses, especially cytokine production and antibody formation, and helps to modify the clinical condition of the patient with rheumatoid arthritis [33].

Bee venom inhibits the DNA-binding activation of NF-kB by inhibiting I-kB phosphorylation. The free and active NF-kB, by the phosphorylation of I kB, is translocated to the nucleus, where it binds to the kB binding sites in the promoter regions of target inflammatory genes and controls their expression.

Thus, our findings are in agreement with those of others showing that the anti-inflammatory activity of honeybee venom is mediated through suppression of the NF-κB signaling pathway [18]. Therefore, increased levels of androgens, IL-6, COX-2 and VEGF can be adjusted by treating rats with HBV for 14 days and bee venom by inhibitory effects on inflammatory index causes regression of PCOS in this animal model [27]. Histological changes observed in the ovary after bee venom treatment may also be considered confirmation of recovery from this syndrome. Our results confirm that bee venom causes a decrease in the follicular theca layer in PCOS rats, which is actually due to decreased ovarian angiogenesis. Due to this decrease, the androgens and steroids produced by this layer also decreased and consequently the total levels of serum estrogen and androgens reduced.

## Conclusions

Based on the results presented in this study, we can conclude that anti-inflammatory effects of HBV can restore metabolic and reproductive features of PCOS. Decreased levels of IL-6, COX-2, VEGF and serum androgens, and increased number of corpora lutea are suggestive of therapeutic effects of HBV on PCOS. We have established that this effect of HBV is mediated by suppression of inflammatory and angiogenic factors.

### Ethics committee approval

All procedures were carried out according to the Guidelines for the Care and Use of Laboratory Animals (National Research Council, 1996).

## Competing interests

The authors declare that they have no competing interests.

## Authors’ contributions

The present work is a collaboration among all authors. Contribution to design analysis, interpretation of data, drafted the article, revised and approved the final version to be published: MN and HMK. Data collection, histological and immunological tests, interpretation of data and drafted the article: LK, HA and AS. Data analysis: AB and LK. Manuscript writing: AS, HA and LK. All authors have contributed to, seen and approved the manuscript.
